# Revealing nonlinear and interaction mechanisms of resource and environmental carrying capacity through explainable machine learning

**DOI:** 10.1371/journal.pone.0360548

**Published:** 2026-10-28

**Authors:** Qianrong Zhang, Yongfeng Zhang, Hongzhen Yu, Feilong Qing, Yao Wang

**Affiliations:** 1 Mingde College, Chengdu Technological University, Chengdu, Sichuan, China; 2 School of Mathematical Science, Chengdu University of Technology, Chengdu, Sichuan, China; 3 Faculty of Information Technology, City University Malaysia, Darul Ehsan, Malaysia; 4 Business College, Jiangxi Institute of Fashion Technology, Nanchang, Jiangxi, China; Zhejiang Agriculture and Forestry University: Zhejiang A and F University, CHINA

## Abstract

Existing studies on resource and environmental carrying capacity (RECC) have primarily focused on comprehensive evaluation and linear identification. But the quantification of driving effect on RECC have not been fully explored, especially the interaction and nonlinear effects. Therefore, this paper constructs an explainable machine learning framework, to comprehensively assess the RECC index of 31 provincial regions in China and to reveal nonlinear and interaction mechanisms of influence factors. Firstly, the XGBoost model is utilized to surrogate the evaluation results, which demonstrates outstanding predictive performance (R^2^ = 0.95, RMSE = 0.08) and stability. The results shows that the average RECC index in China dropping from 1.18 to 0.39, marking a transition from a high-risk state to a safe and sustainable state. Then, the SHAP model quantifies the nonlinear and threshold effects of key influence factors. The results indicate that the total emissions of pollutants in wastewater (*X*_2_) and industrial sulfur dioxide emissions (*X*_3_) are the two most critical factors, with a cumulative contribution rate of 49%. 50000 tons is a threshold for *X*_2_, beyond which the RECC index significantly increases with the increase of *X*_2_. 10 billion cubic meters is the marginal contribution of *X*_3_, with the greatest impact on RECC index. The interaction effect indicates a significant positive synergistic effect between *X*_2_ and *X*_3_, with the maximum increase in SHAP value reaching 0.1. There is a significant antagonistic effect between resource availability and environmental pressure. This study offering new directions for understanding the underlying mechanisms of complex environmental systems.

## Introduction

With the intensification of urbanization and the rapid development of the economy, the pressure on resources and the environment in the region has risen sharply. As the largest developing country, China has achieved remarkable achievements in urbanization and economic development over the past 20 years, with the second-largest GDP in the world. It has also built international cities such as Beijing, Shanghai, and Guangzhou, as well as 19 national urban agglomerations and economic circles. However, the rapid development has also brought about serious resource and environmental problems. In 2022, China’s total energy consumption reached 5.64 billion tons of standard coal, an increase of over 400% compared to 1990, with an average annual growth rate of approximately 4.2%. In 2017, the national industrial wastewater discharge reached about 23.7 billion tons, approximately four times that of 2007. In 2019, China’s carbon emissions totalled approximately 1.4093 billion tons of carbon dioxide equivalent, an increase of over 300% compared to 4.5 billion tons in 2009, with an average annual growth rate of nearly 2% up to 2022. The huge resource consumption and waste emissions have caused severe environmental pollution and ecological damage, posing a huge challenge to China’s sustainable development. This has made it increasingly urgent for China to transform its extensive development model into a sustainable one. Therefore, how to comprehensively enhance the carrying capacity of regional resources and the environment, and reduce the costs of economic restructuring and development model transformation, has become the key role for future economic high-quality development and comprehensive sustainable development.

RECC refers to the population size and economic total that a region can support in terms of resources and the environment during its development process, reflecting the limit of human activities that the ecosystem can bear. As a complex coupled system, early scholars defined RECC include society, economy, resources, and the environment subsystems, emphasizing the interaction, constraint, and balance among the subsystems, including dozens of influencing factors [1,2]. With the continuous deepening of research, more scholars have understood RECC as being coupled by three dimensions: pressure, support, and regulation. Pressure describes the load of human activities on resources and the environment. Support reflects the regional resource supply and ecological guarantee capacity. Regulation reflects the economic investment capacity for resource utilization and environmental protection. Therefore, RECC is not just a static limit value but undergoes dynamic changes following multiple systems and multiple indicators. Thus, to enhance regional resource and environmental carrying capacity, two key issues need to be addressed. On one hand, how to accurately assess the current status of resource carrying capacity and identify its warning line threshold. On the other hand, how to quantify the contribution of influence factors on RECC and find a quantitative collaborative solution to improve carrying capacity.

A scientific evaluation index system is the key to the accuracy of comprehensive assessment. Ma and Duan utilized a variety of spatio-temporal data [3], starting from the perspectives of resources and environment, and designed three main indicators: zoning index, social and economic development index, and population concentration index. These were then used to analyze the carrying capacity of rural areas in China. Peng and Deng examined the issue from three aspects [4]: pressure, support, and regulation, and used this framework to assess the carrying capacity of 31 provincial regions across the country. Wang et al. proposed a more complex “six-party interaction” model, including pressure, support, disruption, recovery capacity, degradation degree, and promoting factors, attempting to more comprehensively reflect the interactions among the systems [5]. Jia and Wang adopted the DPSIR model and combined the TOPSIS method to dynamically analyze the carrying capacity of water resources in Zhengzhou [6]. In general, due to the different research problems and regional characteristics, the evaluation criteria used by everyone are also different.

Traditional statistical methods are commonly used in the study of the driving mechanism of resource and environmental carrying capacity. For example, Sobhani and Esmaeilzadeh found that the impact of natural resources on carrying capacity varies greatly in different places and management methods when studying the Jajurut Nature Reserve in Iran using the Ecological Stress Index [7]. Bole and Rina focus on western Jilin and find that population density is the number one factor that crushes the local resource and environmental carrying capacity [8]. In addition to traditional statistics, some researchers also use more complex mathematical models to uncover deeper reasons. Using a dynamic model that can simultaneously observe temporal and spatial changes to analyze the water resources carrying capacity in Xinjiang, it was found that in the early years, natural ecological conditions were mainly dominant, but later it gradually shifted to social factors such as economy and population playing a dominant role [9]. Cheng and Huang attempted to use the machine learning method of “random forest” to identify key variables that affect ecological carrying capacity [10], and combined it with exponential smoothing to predict future trends, pointing out that ecological degradation and economic development are the two core driving forces. When studying the Yellow River Basin, Cheng and Zhu used the LMDI decomposition model and found that improving wastewater treatment capacity and technological progress can effectively enhance the carrying capacity of water resources [11]. However, economic growth actually reduces the carrying capacity. In other words, relying solely on economic development without paying attention to environmental protection will make the water environment even tighter. Overall, these studies indicate that the factors affecting carrying capacity are not fixed and need to be combined with specific regions and development stages in order to identify the root cause of the problem.

Overall, current research on resource and environmental carrying capacity and its influence factors mostly relies on traditional indicator systems and statistical methods [12]. But these methods have a clear weakness, as they often rely on expert scoring or simply weighted averages of various indicators, which cannot accurately quantify the nonlinear relationship between influencing factors in multiple systems, such as the trend of a single factor’s impact on result changes, or how multiple factors affect each other and work together [13,14]. Moreover, traditional statistical methods are inadequate in dealing with the complex relationships between variables. Often, they can only roughly determine which factor is important, but cannot explain how much impact it has, at what critical point it will trigger a mutation, or perform sensitivity analysis well. Although methods such as ecological footprint can roughly show the trend relationship between carrying capacity and human activities, both traditional statistics and ecological footprint are difficult to accurately calculate the marginal contribution of a influence factor. In other words, existing methods are qualitatively acceptable, but there are still significant shortcomings in quantitatively characterizing the true operating mechanisms of complex systems.

To address these shortcomings, machine learning methods have brought new ideas due to their expertise in handling complex and nonlinear relationships [15]. Compared with traditional statistical methods, machine learning not only predicts more accurately and has stronger adaptability, but also does not rely on subjective scoring by experts or manually setting weights, and can directly “dig” out the complex relationships hidden between variables from the data [16]. Many research achievements have been made, such as Zhang and Li’s evaluation of water resources carrying capacity in Liaoning Province [17], which simultaneously tested several models including Random Forest (RF), Support Vector Machine (SVM), Multi Layer Perceptron (MLP), and K-Nearest Neighbor (KNN). The results showed that Random Forest had the best performance. But most machine learning models are like a ‘black box’, although their predictions are accurate, they cannot explain exactly which factors are at work and how they work. This leads to it being able to simulate the carrying capacity level of a place, but unable to explain the driving mechanism behind it. To solve this problem, researchers have started using SHAP (Shapley Additive Explanation) method, which can make the model more “transparent” while maintaining high prediction accuracy. By using tools such as SHAP values, feature importance ranking, and partial dependency graphs, not only can key influencing factors be clearly identified, but also how they affect bearing capacity can be analyzed, and even critical points of action can be identified [18]. Based on this idea, this article constructs an interpretable machine learning framework that combines XGBoost and SHAP to comprehensively evaluate the resource and environmental carrying capacity of various provinces in China, and deeply analyze the driving mechanisms. The main research topics include:

(1) XGBoost as a nonlinear surrogate model for TOPSIS evaluation results. First, the Technique for Order Preference by Similarity to Ideal Solution (TOPSIS) combined with entropy weights was employed to conduct a comprehensive assessment of the resource and environmental carrying capacity across China’s 31 provinces. Subsequently, the XGBoost model was utilized as a surrogate for the assessment results to capture the complex nonlinear coupling relationships among the indicators. Meanwhile, a spatio-temporal evolution analysis of the resource and environmental carrying capacity was performed from both temporal and spatial dimensions.
(2) Demonstrate the superiority of the XGBoost model. Based on the evaluation results of resource and environmental carrying capacity, six classical machine learning models were established: MLP, KNN, SVM, RF, LM, and XGBoost. The performance of these six models was compared across evaluation metrics including MSE, RMSE, MAE, and R^2^. The results validated that the XGBoost model outperforms the other machine learning models.
(3) XGBoost combines with SHAP to quantify the model contribution of key factors to the RECC index. The importance of influence factors is quantified through the average SHAP value of each variable. The marginal contribution and threshold effects of primary influence factors on RECC index are quantified via single-factor SHAP dependency plots. The interactive influence of primary influence factors on RECC index is quantified through SHAP interaction dependency plots.

## Methodology

### Data source

This paper compiles resource and environmental data from China’s 31 provinces and regions spanning 2010 to 2022. Due to severe data gaps, the research discussion excludes Hong Kong, Macao, and Taiwan. Basic information, such as administrative area size, population growth rate, and GDP, is sourced from the China Statistical Yearbook (Source: https://www.stats.gov.cn). Energy information is sourced from the China Energy Statistical Yearbook (Source: https://www.las.ac.cn), including indicators such as *X*_6_ and *X*_11_. Urban development data is sourced from the China Urban Statistical Yearbook (Source: https://www.mohurd.gov.cn), including indicators such as *X*_9_ and *X*_10_. Environmental protection information is sourced from the China Ecological and Environmental Status Bulletin (Source: https://www.mee.gov.cn), such as *X*_2_, *X*_3_.

For the Tibet region, missing data for *X*_11_, *X*_6_, and other variables from 2010 to 2015 were imputed using polynomial interpolation, as shown in Formula 1. PM2.5 data was missing from 2010 to 2012. Observing the data trend, it followed a linear pattern, so linear interpolation was used for filling. As shown in Formula 2. To unify the data scale and eliminate the influence of data dimensions on the model, all data underwent normalization processing. Formula 3 represents the normalization of positive indicators, and Formula 4 represents the normalization of negative indicators.


$$\begin{array}{c} y=a_{k}x^{k}+a_{k-1}x^{k-1}+\cdots +a_{\text{0}} \end{array}$$ (1)

Here, *k* denotes the degree, a represents the coefficient, and (*x*, *y*) indicates the inserted data point.


$$\begin{array}{c} y-y_{\text{0}}=k_{\text{0}}\left(x-x_{\text{0}}\right) \end{array}$$ (2)

Where (*x*, *y*) represents the inserted data point, $\left(x_{\text{0}},y_{\text{0}}\right)$ denotes the nearest data point. k0 is the rate of change between the average of all points and the nearest point.


$$\begin{array}{c} a_{ij}=\frac{x_{ij}-min\;x_{ij}}{max\;x_{ij}-min\;x_{ij}} \end{array}$$ (3)

$$\begin{array}{c} a_{ij}=\frac{max\;x_{ij}-x_{ij}}{max\;x_{ij}-min\;x_{ij}} \end{array}$$ (4)

Among these, *a*_*ij*_ represents the normalized value, falling within the range [0,1]. $x_{ij}$ represents the original value of indicator *j*.

### Establish the RECC indicator system

Carrying capacity originally refers to a concept in mechanics, denoting the maximum load an object can withstand without sustaining any damage [19]. Scholars have integrated this concept with ecological environments, resources, and economic development to propose ecological carrying capacity, resource carrying capacity, and economic carrying capacity [20]. Resource and environmental carrying capacity represents the combined manifestation of resource carrying capacity and environmental carrying capacity, constituting a complex coupled system. Different scholars have interpreted resource and environmental carrying capacity from diverse perspectives [21]. This paper argues that, in addition to pressure and carrying capacity, regulatory forces stemming from socioeconomic progress in resource utilization, pollution control, and ecological conservation should also be considered. Therefore, this study adopts a resource-environment carrying capacity evaluation framework based on pressure-carrying capacity-regulatory forces, comprising 18 evaluation indicators as shown in Table 1.

**Table 1:** Indicator system based on pressure-supporting force-regulating force.

Target Layer	Indicator	Calculation Formula	Unit
Pressure	Total water consumption (*X*_1_)	Sourced from the China Statistical Yearbook	10^8^ m^3^
	Total pollutant discharge in wastewater (*X*_2_)	The total amount of harmful metal elements in wastewater	ton
	Industrial sulfur dioxide emissions (*X*_3_)	Sourced from the China Environmental Status Report	10^4^ ton
	Annual average concentration of PM2.5 (*X*_4_)	Sourced from the China Environmental Status Report	μg/m^3^
	Population density (*X*_5_)	Total population/ Total area	person/km^2^
	Total energy consumption (*X*_6_)	Sourced from the China Energy Statistical Yearbook	10^4^ ton
Support	Total Water Resources Volume (*X*_7_)	Sourced from the China Energy Statistical Yearbook	10^8^ m^3^
	Cultivated land per capita (*X*_8_)	Total cultivated land/Total population	m^3^/person
	Green land per capita (*X*_9_)	Total green land/Total population	m^3^/person
	Forest coverage rate (*X*_10_)	Total forest/Total area	%
	Total energy production (*X*_11_)	Sourced from the China Energy Statistical Yearbook	10^4^ ton
Regulatory capacity	GDP per capita (*X*_12_)	Sourced from the China Statistical Yearbook	yuan
	Wastewater treatment rate (*X*_13_)	Total wastewater treatment/Total wastewater	%
	Waste disposal volume per capita (*X*_14_)	Total waste disposal/Total population	%
	Tertiary industry proportion (*X*_15_)	GDP of tertiary industry/GDP	%
	Environmental expenditure proportion (*X*_16_)	Environmental expenditure/Total expenditure	%
	Number of patents (*X*_17_)	Sourced from the China Statistical Yearbook	unit
	Resource development expenditure proportion (*X*_18_)	Resource development expenditure/Total expenditure	%

The pressure component selected six indicators. *X*_1_ and *X*_6_ reflect the consumption of resources such as water, electricity, coal, and oil in the region, describing the current state of resource pressure. *X*_5_ describes the level of local social activities and urbanization, depicting the current state of social pressure. *X*_2_, *X*_3_, and *X*_4_ reflect the environmental conditions of the region, illustrating the current state of environmental pressure.

The carrying capacity component comprises five indicators. *X*_7_ reflects the region’s freshwater supply capacity, serving as a foundational environmental resource supporting population and economic activities while directly determining the water resource limits of carrying capacity. *X*_8_ measures per capita available agricultural production space. *X*_9_ reflects ecological environment quality and livability. *X*_10_ reflects the vegetation coverage level of the overall ecosystem, representing carbon sequestration capacity, ecological restoration potential, and buffering effects against environmental pressures. These are crucial indicators for assessing environmental support capacity. *X*_11_ characterizes the region’s energy self-sufficiency, serving as a key resource for maintaining resource carrying capacity levels and ensuring sustainable development.

The regulatory capacity component comprises seven indicators. *X*_12_ measures regional economic development levels and regulatory capabilities, providing the economic foundation for environmental governance and resource utilization. *X*_15_ reflects the degree of economic structural optimization, with the tertiary sector typically bearing lower environmental burdens, thereby alleviating resource and environmental pressures. *X*_13_ and *X*_14_ evaluate environmental governance capacity, which is crucial for optimizing environmental quality and regulating carrying capacity. *X*_16_ measures the government’s willingness and ability to invest financially in environmental protection, reflecting the priority given to green development. *X*_18_ indicates the level of investment in protection and restoration during resource development and utilization, helping to enhance resource efficiency and reduce ecological losses. *X*_17_ assesses innovation capability, representing the potential for green technology development and serving as a key driver for improving resource efficiency and environmental management standards.

### Building comprehensive evaluation model

Resource and Environmental Carrying Capacity (RECC) encompasses both resource carrying capacity and environmental carrying capacity, and is inextricably linked to resources, the environment, society, and the economy. Therefore, at its core, the evaluation of resource and environmental carrying capacity is the result of coupling and coordination among multiple systems and indicators [22]. The TOPSIS method objectively reflects the comprehensive performance of China’s 31 provinces in terms of resource and environmental pressure, support capacity, and regulatory capacity by calculating the proximity of each evaluation object to the ideal state. Consequently, this paper applies the TOPSIS method to the comprehensive evaluation of resource and environmental carrying capacity at the provincial level in China. The calculation process is as follows.

#### Initialize the subsystem matrix.

Create a standardized initial matrix *R*_*i*_ for each of the pressure subsystem, support force subsystem, and adjustment force subsystem. This matrix is used to calculate subsequent weights and the overall score, as shown in Equation 5.


$$\begin{array}{c} R_{i}=\left[\begin{array}{ccc} a_{11} & \cdots & a_{1m}\\ \vdots & \ddots & \vdots \\ a_{n1} & \cdots & a_{nm} \end{array}\right] \end{array}$$ (5)

Where, *a*_*ij*_ denotes the standardized value of indicator *j* for city *i*, m represents the total number of indicators for each subsystem, and *n* indicates the total sample size.

#### Weight calculation.

The entropy weight method is employed to calculate the weight of each evaluation indicator. It quantifies the dispersion of indicators by computing their information entropy, thereby determining their respective weights [23]. As shown in Equations (6)–(8).


$$\begin{array}{c} p_{ij}=\frac{a_{ij}}{\sum_{i=1}^{n}a_{ij}} \end{array}$$ (6)

$$\begin{array}{c} e_{j}=-\frac{\text{1}}{ln\left(n\right)}\sum_{i=1}^{n}p_{ij}\;ln\left(p_{ij}\right) \end{array}$$ (7)

$$\begin{array}{c} w_{j}=(1-e_{j})/\sum_{j=1}^{m}(1-e_{j}) \end{array}$$ (8)

Where, $P_{ij}$ denotes the relative contribution of each sample point to indicator *j*. *e*_*j*_ represents the information entropy of evaluation indicator *j*. *w*_*j*_ indicates the weight of indicator *j*.

#### Calculating comprehensive index of subsystem.

The comprehensive indices for the pressure subsystem, support force subsystem, and adjustment force subsystem were calculated using the TOPSIS method [24]. The calculation process is detailed in Equations (9)–(11).


$$\begin{array}{c} Z_{i}^{k}=\frac{D_{i}^{-}}{D_{i}^{+}+D_{i}^{-}},k=1,2,3 \end{array}$$ (9)

$Z_{i}^{k}$ represents the composite index of the three subsystems, with values ranging from 0 to 1.

$D_{\text{i}}^{+}$ indicates the distance from the positive ideal solution, $D_{\text{i}}^{-}$ represents the distance from the negative ideal solution.


$$\begin{array}{c} D_{i}^{+}=\sqrt{\sum_{j=1}^{m}w_{j}(a_{ij}-a_{j}^{+})} \end{array}$$ (10)

$$\begin{array}{c} D_{i}^{-}=\sqrt{\sum_{j=1}^{m}w_{j}(a_{ij}-a_{j}^{-})} \end{array}$$ (11)

#### Calculating comprehensive index of RECC.

Within the pressure-support-regulation framework, RECC is a complex nonlinear system. It simultaneously describes the negative impact of pressure on RECC while reflecting the positive effects of support and regulation on RECC. Therefore, this study employs a linear ratio method to calculate the comprehensive index of water resource carrying capacity (Equation12).


$$\begin{array}{c} RECCI=\frac{P}{S+R} \end{array}$$ (12)

Among these, P, S, and R represent the pressure index, support force index, and regulation force index, respectively. RECCI represents the comprehensive index of a region’s resource and environmental carrying capacity. A higher value indicates a lower RECC, signifying that resource and environmental pressures significantly exceed the region’s support and regulatory capacities. Conversely, a lower value indicates a higher RECC, indicating that the region’s resource and environmental support and regulatory capacities are fully capable of accommodating the pressures brought by development.

To better analyze the risk levels of resource and environmental carrying capacity. we have categorized the comprehensive index IRECC. Using RECCI = 1 as the threshold, it is divided into four categories: the first category is the safe zone, where RECCI <0.5. Pressure is significantly lower than the sum of carrying capacity and regulatory capacity, indicating that the resource and environmental pressures generated by development in a region remain entirely within tolerable limits. Second, the cautionary zone 0.5 < RECCI < 0.75 indicates that carrying capacity and regulatory capacity are relatively well-equipped to withstand pressures, but risks of approaching dangerous levels exist. Third, the Danger Zone 0.75 < RECCI < 1 indicates that carrying capacity is approaching or has reached a critical state. This signifies significant resource and environmental pressures or insufficient support and regulation capacities, necessitating adjustments based on limiting factors to prevent exceeding critical thresholds. Fourth is the imbalance zone, RECCI > 1,indicating extremely low resource-environmental carrying capacity and unsustainable development requiring accelerated adjustments. Concurrently, blue warning thresholds (RECCI = 0.5), yellow warning thresholds (RECCI = 0.75), and red warning thresholds (RECCI = 1). Classification criteria are detailed in Table 2.

**Table 2:** Resource and environmental carrying capacity classification intervals.

Interval	RECC Level	Interval	RECC Level
(0, 0.5)	Safe Zone	0.5	Blue warning line
(0.5, 0.75)	Caution Zone	0.75	Yellow warning line
(0.75, 1)	Danger Zone	1	Red warning line
(1, +∞)	Imbalance Zone		

### XGBoost surrogate model

The entropy-weighted TOPSIS method provides an observed composite RECC index. However, the mathematical aggregation process cannot reveal the nonlinear relationships among environmental drivers. Therefore, XGBoost is employed as a surrogate learning model to approximate the mapping:


$$\begin{array}{c} RECCI=f\left(X_{\text{1}},X_{\text{2}},\cdots ,X_{18}\right) \end{array}$$ (13)

where *RECCI* denotes the observed RECC index and *X*_i_ denotes environmental drivers.The purpose of XGBoost is not to reconstruct the TOPSIS results, but to learn the underlying nonlinear relationships embedded in the multidimensional indicator system.

XGBoost is an ensemble learning algorithm that typically employs decision trees as base classifiers. It trains multiple weak learners iteratively, then combines their learning outcomes to form a strong learner, yielding more accurate predictions [25]. Its ability to dynamically adjust parameters enables the model to better handle nonlinear and complex coupled data. RECC involves the coupling of multiple systems and indicators. Utilizing XGBoost enables the identification of RECC coupling mechanisms directly from the data level within vast information sets [26]. The computational process is illustrated in Equation 14.


$$\begin{array}{c} \hat{y_{i}}=\sum_{k=1}^{K}f_{k}\left(X\right),f_{k}\subseteq F \end{array}$$ (14)

Among these, $\hat{y_{i}}$ represents the predicted resource-environment carrying capacity index. *X* denotes the vector set of influence factors, *f*_*k*_ refers to a single decision tree, *K* signifies the total number of decision trees, and *F* constitutes a space composed of weak learners.

The model training process is achieved by minimizing the objective function, which comprises both a loss function and a regularization term. The loss function measures the mean squared error between the predicted RECC index and the evaluated RECC index, while the regularization term controls model complexity to prevent overfitting. The objective function is defined as in Equation 15.


$$\begin{array}{c} L=\sum_{i=1}^{n}l\left(y_{i},\hat{y_{i}}\right)+\sum_{k=1}^{K}\Omega \left(f_{k}\right) \end{array}$$ (15)

Here, *l* denotes the mean squared error, and Ω denotes the complexity.

To evaluate the model’s performance, four metrics were selected: coefficient of determination (R^2^), mean squared error (MSE), root mean squared error (RMSE), and mean absolute error (MAE). The calculation process is detailed in Equations (16) to (19).


$$\begin{array}{c} R^{\text{2}}=1-\sum_{i=1}^{n}(\hat{y_{i}}-y_{i}{)}^{\text{2}}/\sum_{i=1}^{n}(\overset{―}{y_{i}}-y_{i}{)}^{\text{2}} \end{array}$$ (16)

$$\begin{array}{c} MSE=\frac{\text{1}}{n}\sum_{i=1}^{n}(y_{i}-\hat{y_{i}}{)}^{\text{2}} \end{array}$$ (17)

$$\begin{array}{c} RMSE=\sqrt{\frac{\text{1}}{n}\sum_{i=1}^{n}(y_{i}-\hat{y_{i}}{)}^{\text{2}}} \end{array}$$ (18)

$$\begin{array}{c} MAE=\frac{\text{1}}{n}\sum_{i=1}^{n}\left|y_{i}-\hat{y_{i}}\right| \end{array}$$ (19)

Among these, $\hat{y_{i}}$ represents the predicted value of the RECC index, $y_{i}$ represents the actual value, $\overset{―}{y_{i}}$ represents the average value, and n denotes the total number of evaluation subjects.

### Shap model

Although the XGBoost model can accurately predict the Resource and Environmental Carrying Capacity (RECC) index of each province, it is like a black box. We can see the results, but we are not sure how it is calculated. To address this issue, we introduced the SHAP model, which is a specialized interpretability method designed to “open the black box” and is often used in conjunction with machine learning or deep learning models. The basic idea of SHAP is to calculate the additional contribution of each factor in the model prediction, that is, how much it improves or deteriorates the results after being added, and then take the average to measure how important this factor is [27]. In this way, we can not only know which factors are most critical, but also see how they dynamically affect RECC at different values, such as when the pollutant discharge reaches how many tons, the carrying capacity will suddenly decrease. This analysis is particularly useful for understanding the operating mechanism behind RECC, and can even help us identify specific management “critical points” or “thresholds”. The specific calculation method of SHAP value is shown in formula 20 [28].


$$\emptyset_{i}\sum_{S\subseteq X\backslash \left\{i\right\}}\frac{\left|S\right|!\left(\left|X\right|-\left|S\right|-1\right)!}{\left|X\right|!}\left[f_{X}\left(S\cup \left\{i\right\}\right)-f_{X}\left(S\right)\right]$$ (20)

Here, $\phi_{i}$ denotes the SHAP value for the *i*-*th* driver. *X* represents the set of all drivers, while *S* denotes the set excluding driver *i*. This indicates the model’s output value when driver *i* is not considered. $f_{x}\left(S\right)$ denotes the model’s output value when driver *i* is not considered. $f_{x}(S\cup \{i\})$ denotes the model’s output value when driver *i* is incorporated. $f_{x}(S\cup \{i\})-f_{x}\left(S\right)$ denotes the marginal contribution to the output when adding driver *i*. The sum of SHAP values for all features and the initial expected value constitutes the model’s prediction result [29]. As shown in Formula 21.


$$\begin{array}{c} f\left(x\right)=f(\emptyset )+\sum_{i=1}^{m}\phi_{i} \end{array}$$ (21)

Here, f(∅) is a constant representing the mean value when the model has no information input. *m* denotes the total number of influence factors.

### Summary

This paper building an explainable learning framework using XGBoost and SHAP model, which is assessed the resource and environmental carrying capacity across Chinese provinces from 2010 to 2022. Meanwhile, quantifying the model contribution of key influencing factors on RECC index. The overall framework encompasses three aspects. Step one, comprehensive evaluation and temporal and spatial evolution analysis of RECC index. Building an indicator system based on pressure, support, and regulation force. Combining TOPSIS and entropy weighting, the RECC index for each Chinese province are assessed. Spatial trend maps and spatial evolution maps were utilized to analyze the spatio-temporal evolution trends of RECC index. Step two, XGBoost serves as surrogate model to learn the nonlinear mechanism that the TOPSIS model lacks. And comparing the performance of six machine learning models. The optimal machine learning model was identified across four evaluation metrics. Concurrently, k-fold cross-validation and variable perturbation methods confirmed the XGBoost model’s superior sensitivity and stability. Step three, Quantifying the marginal contributions and threshold effects of influence factors. Variable importance was analyzed using SHAP plots and dependency maps. Single-factor SHAP dependency maps were employed to interpret the nonlinear impacts of primary drivers. SHAP interaction dependency maps were used to explain the interactive effects and threshold effects of influence factors on RECC index. The overall framework is illustrated in Fig 1.

**Fig 1:**
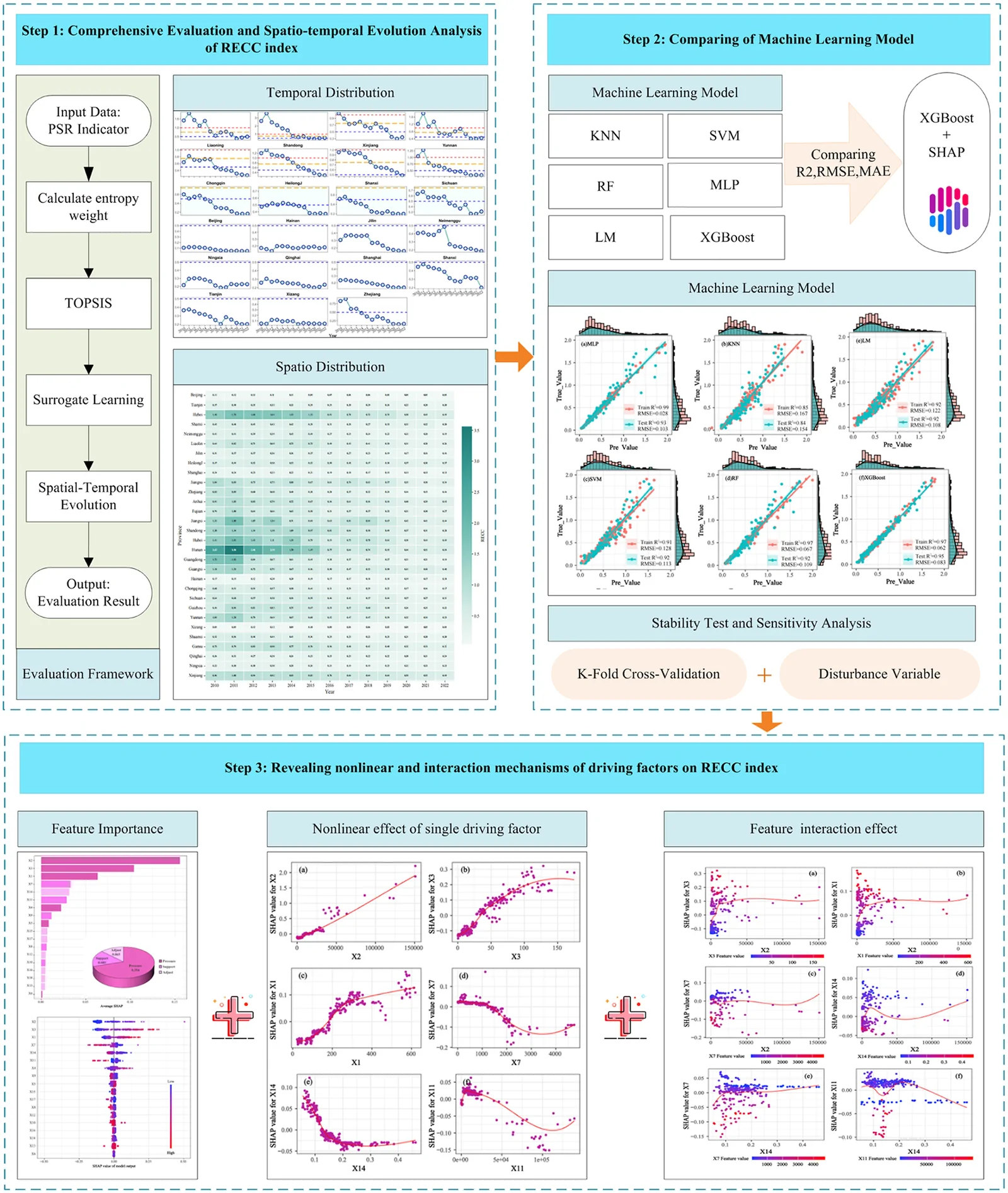
Overall research framework.

## Results and analysis

### RECC index spatio-temporal evolution

To better analyze the temporal evolution trends of resource and environmental carrying capacity across provinces, blue, orange, and red warning thresholds have been established. Based on the comprehensive scores of each province’s resource and environmental carrying capacity, they are categorized into three groups: The first group exhibits very strong carrying capacity, consistently remaining below the blue warning threshold annually. The second group demonstrates moderate carrying capacity, with some years falling between the orange and blue warning thresholds. The third group indicates significant risk, with capacity exceeding the red warning line in some years.

The resource and environmental carrying capacity index of each province in China from 2010 to 2022 can be roughly divided into three categories (Fig 2). The first category includes 10 provinces and autonomous regions, including Beijing, Hainan, Jilin, and Inner Mongolia. The overall score is lower than 0.5 (below the blue warning line) for a long time, and Beijing, Hainan, Xizang, and other places even lower than 0.2 for a long time. For example, Qinghai, Ningxia, Xizang and other places, due to small industrial scale and low resource consumption, the pressure is not great; Although Beijing and Shanghai have high development intensity, they invest heavily in environmental protection and have high resource utilization efficiency. Therefore, they have strong support and regulation capabilities, and instead have high carrying capacity, becoming benchmarks for green development. The second category is Heilongjiang, Shaanxi, Sichuan, and Chongqing, which are partially between the blue and orange warning lines in some years: Heilongjiang briefly “overloaded” from 2011 to 2016 and then returned to safety; Shaanxi only slightly exceeded the limit in 2010–2011; Sichuan and Chongqing experienced significant fluctuations between 2010 and 2015, but have steadily improved and entered a safe zone since 2016. The third category covers 17 provinces including Anhui, Fujian, and Gansu, where early problems were more prominent. From 2010 to 2014, all provinces exceeded the orange warning line, with heavy industrial provinces such as Hunan (2.62) and Henan (1.71) even breaking through the red warning line. After 2015, there was a significant improvement. In 2016, all provinces withdrew from the red zone, with most leaving the orange zone. Zhejiang (0.28) and Fujian (0.31) were the first to achieve sustainable development; From 2019 to 2022, with further improvement, 11 provinces have entered the blue safety line, with Zhejiang (0.61) and Guangdong (0.19) leading the country, and only 6 provinces including Henan (0.50) and Hubei (0.49) still hovering on the edge of the safety line.

**Fig 2:**
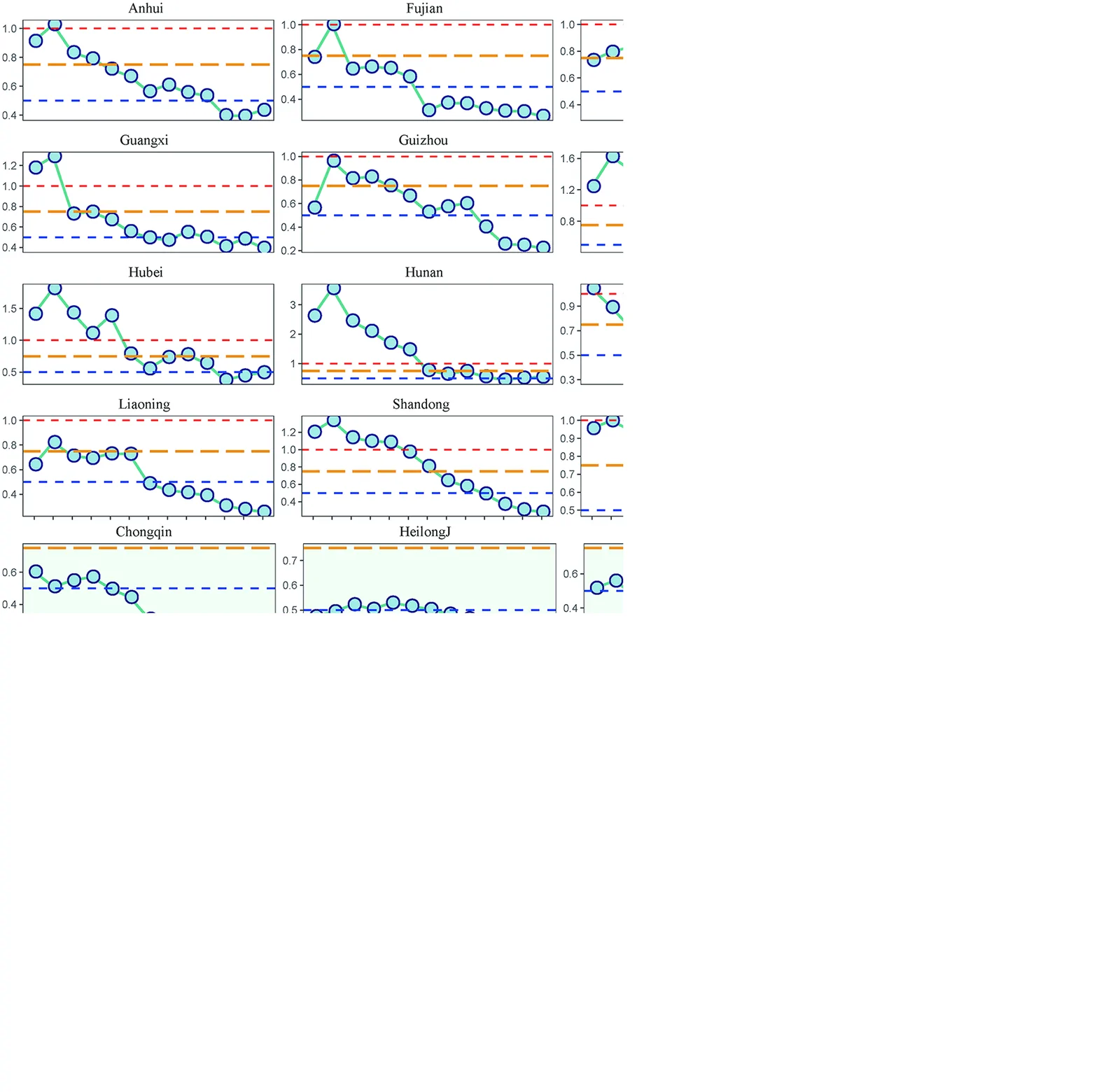
The temporal distribution of RECC from 2010 to 2022.

The overall trend shows significant improvement in China’s provincial RECC from 2010 to 2022, with the average RECC index decreasing from 1.18 in 2010 to 0.39 in 2022. All provinces have moved away from the red warning line. The number of regions entering the safe and sustainable development zone increased from zero to eleven.

The spatial evolution of the RECC index for 31 cities is depicted in Fig 3, which exhibits the following characteristics.

**Fig 3:**
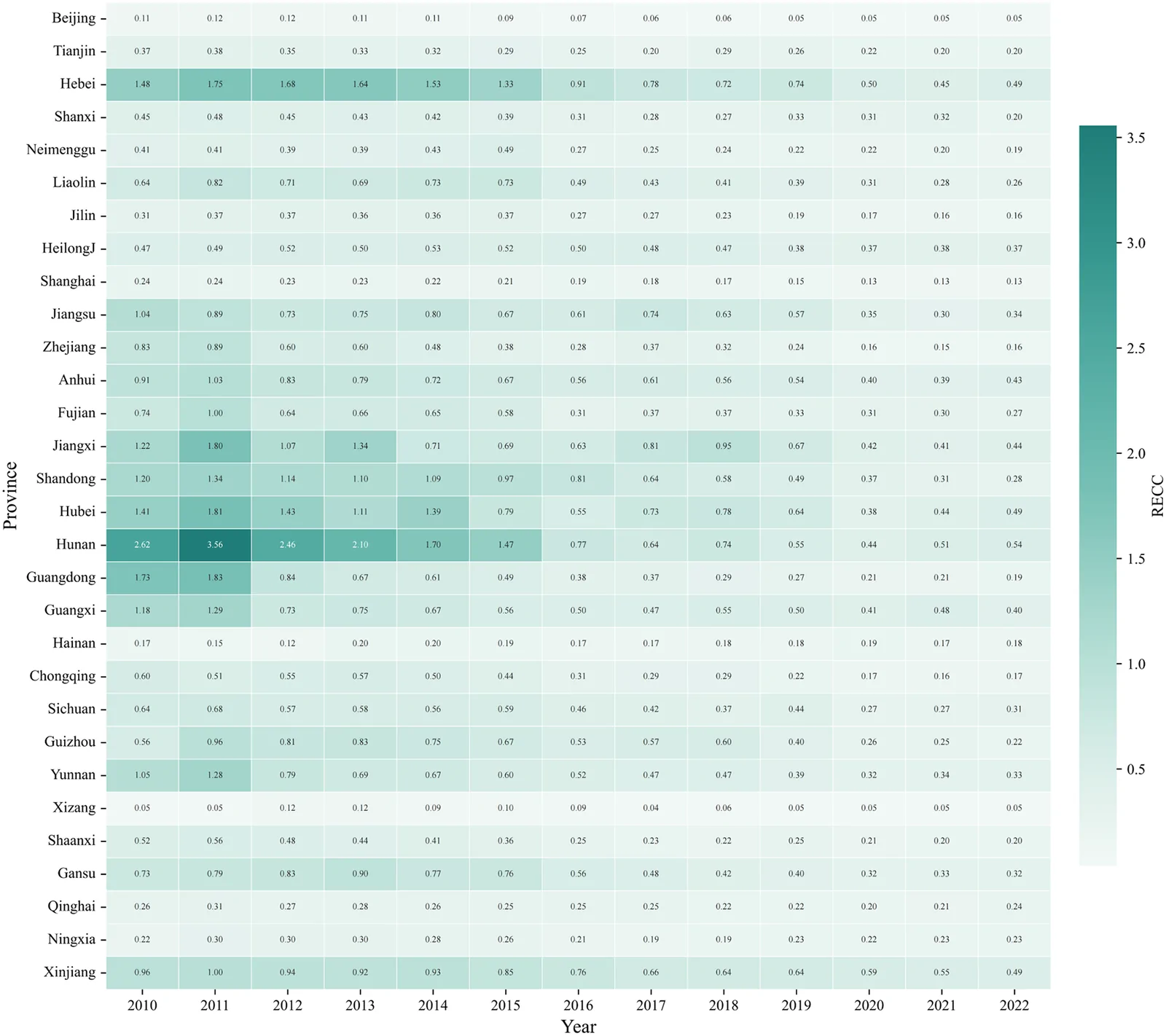
The spatial evolution of the RECC.

First. High-risk areas have shifted from concentrated clusters to scattered distribution. In 2010, high-risk regions were primarily concentrated in North China (Hebei, Shandong), Central China (Henan, Hubei, Hunan), East China (Jiangsu, Jiangxi), and South China (Guangdong, Guangxi, Yunnan). By 2013, they were concentrated in North and Central China. By 2015, they were only scattered in areas such as Hebei, Hunan, and Hubei. By 2022, no provinces nationwide were classified as high-risk. Second, regional disparities gradually narrowed, though central regions lagged relatively behind. Eastern coastal provinces showed the most rapid improvement, with Guangdong dropping from 1.73 (2010) to 0.19 (2022) and Zhejiang falling from 0.83 to 0.16. Although central regions showed significant improvement. Hunan’s ratio dropped from 2.62 to 0.54. Henan, Hubei, and Hunan remained within the orange alert range (0.5–0.75) in 2022, while most eastern and western provinces had entered the safe zone. Western regions demonstrated overall marked improvement, though some provinces (Gansu, Xinjiang) initially progressed slowly before gradually entering the safe zone. Third, the spatial pattern has shifted from “overall danger in the central region” to “safety and sustainability in the central region.” In 2010, all six central provinces exceeded the orange alert threshold, with five surpassing the red alert threshold, classifying them as high-risk areas. By 2015, only Henan and Hunan remained high-risk areas in the central region. By 2022, all six central provinces had dropped below 0.75, with only Henan, Hubei, and Hunan still within the orange alert threshold (0.5–0.75), classified as caution zones. Meanwhile, most areas in the eastern and western regions entered the safe zone (<0.5).

Overall, China’s provincial resource and environmental carrying capacity has seen a general improvement. Spatial evolution exhibits the following characteristics: “Southwest and Northeast regions have maintained coordinated development while gradually enhancing their capacity. Eastern regions initially showed weak carrying capacity but rapidly strengthened it. Central regions experienced extreme imbalance in early stages but have achieved sustained improvement.”

### Machine learning model comparison

#### Comparison of evaluation indicators.

This article develops an integrated machine learning framework based on the pressure support regulation framework for comprehensive evaluation of resource and environmental carrying capacity. Six classic machine learning models, XGBoost, KNN, SVM, RF, LM, and MLP, were selected. By comparing the performance indicators of the models in the training and testing sets, the most suitable machine learning surrogate model for the resource and environmental carrying capacity of Chinese provinces was found.

The dataset contained 403 observations, of which approximately 282 observations (70%) were randomly assigned to the training set and 121 observations (30%) to the test set. With RECC as the continuous target variable and *X*_1_ to *X*_18_ as the input variables, six machine learning models were established. In terms of parameter settings, the XGBoost model was set with a fixed learning rate of 0.1, a tree depth of 3, and 100 iterations. The other five models were based on the caret framework, using 10-fold cross-validation, and underwent centering and standardization preprocessing. The hyperparameters were automatically searched through tuneLength = 10, namely KNN searched for the number of neighbors K = 10, SVM searched for the cost parameter C = 10, Random Forest searched for the number of split node variables mtry = 10, Multi-Layer Perceptron (MLP) searched for the number of hidden layer nodes and weight decay coefficient. Linear regression did not require setting hyperparameters. At the same time, the random seed number was set to 101.

Table 3 describes the performance metrics of six machine learning models in the training and testing sets, including MSE, RMSE, MAE, and R^2^. In the training set, the R^2^ of all 6 machine learning models reached above 0.85, with MLP and XGBoost models being the best, with R^2^ values of 0.99 and 0.97, respectively. MSE and RMSE performed well, with MLP and XGBoost being the best. The MAE of the six models are all less than 0.1, with MLP, RF, and XGBoost being better, all less than 0.05. In the test set, R^2^ is also above 0.85, with XGBoost (0.95) and MLP (0.93) being the optimal values. MSE and RMSE are only above 0.15 for the KNN model, while the rest of the models are around 0.1. XGBoost (0.08) and MLP (0.1) are optimal. MAE is below 0.1, and XGBoost and MLP are both 0.06, which is the best. Through comparative analysis, it can be concluded that MLP and XGBoost models perform the best in both the training and testing sets. MLP outperforms XGBoost in the training set, while XGBoost outperforms MLP in the testing set, with a larger R^2^ and smaller RMSE.

**Table 3:** Evaluation index of six machine learning model.

Data Set	Type	MLP	KNN	SVM	RF	LM	XGBoost
Training Data	MSE	0.0008	0.028	0.0164	0.0045	0.0150	0.0039
	RMSE	0.0286	0.1673	0.1282	0.0667	0.1224	0.0627
	MAE	0.0204	0.0946	0.0808	0.0310	0.0843	0.0450
	R^2^	0.9958	0.8564	0.9156	0.9772	0.9231	0.9798
Testing Data	MSE	0.0107	0.0239	0.0124	0.0120	0.0117	0.0070
	RMSE	0.1034	0.1546	0.1113	0.1094	0.1084	0.0839
	MAE	0.0612	0.0934	0.0774	0.069	0.0822	0.0640
	R^2^	0.9314	0.8466	0.9205	0.9233	0.9246	0.9501

To test the uncertainty of the XGBoost model, the Bootstrap resampling method was adopted. The number of resampling iterations was set to 200, and the mean values and 95% confidence intervals of the evaluation metrics MSE, RMSE, MAE, and R2 were calculated (Table 4). Among them, the 95% confidence interval of R2 was [0.86, 0.95], and the RMSE was [0.08, 0.24]. These results indicate that the XGBoost model maintains relatively strong and stable predictive performance under repeated Bootstrap resampling.

**Table 4:** The 95% confidence interval estimation result of the XGBoost model.

Evaluation index	Mean		95% Confidence Interval	
	Training	Testing	Training	Testing
MSE	0.0007	0.0263	[0.0005, 0.0008]	[0.0076, 0.0617]
RMSE	0.0261	0.1623	[0.0229, 0.0289]	[0.0871, 0.2485]
MAE	0.0189	0.0788	[0.0166, 0.0211]	[0.0578, 0.1073]
R^2^	0.9953	0.9013	[0.9934, 0.9966]	[0.8614, 0.9501]

The scatter plots of predicted and observed values and their edge density distributions (Fig 4) were used to evaluate the performance of six machine learning models. At the same time, their coefficient of determination (R^2^) and root mean square error (RMSE) were compared. MLP, KNN, and SVM models have significant shortcomings. MLP shows moderate predictive ability (Test set R^2^ = 0.92, RMSE = 0.12), but there is a risk of overfitting (Training set R^2^ = 0.99, RMSE = 0.028). KNN showed a wide error band on both the training and testing sets (Training set R^2^ = 0.85, RMSE = 0.16; Test set R^2^ = 0.84, RMSE = 0.15) indicates that the model’s prediction accuracy is insufficient and there is an issue of underfitting. Random forest shows high accuracy (Training set R^2^ = 0.97, RMSE = 0.06; Test set R^2^ = 0.92, RMSE = 0.10) reflects its robust fitting ability and good generalization performance. Linear model (Training set R^2^ = 0.92, RMSE = 0.12; Test set R^2^ = 0.92, RMSE = 0.10) and SVM model (Training set R^2^ = 0.91, RMSE = 0.12; Test set R^2^ = 0.92, RMSE = 0.11) has a high overall predictive ability, but the residual variance at both ends of the numerical interval is large, indicating that it is difficult to capture nonlinear patterns in the data.

**Fig 4:**
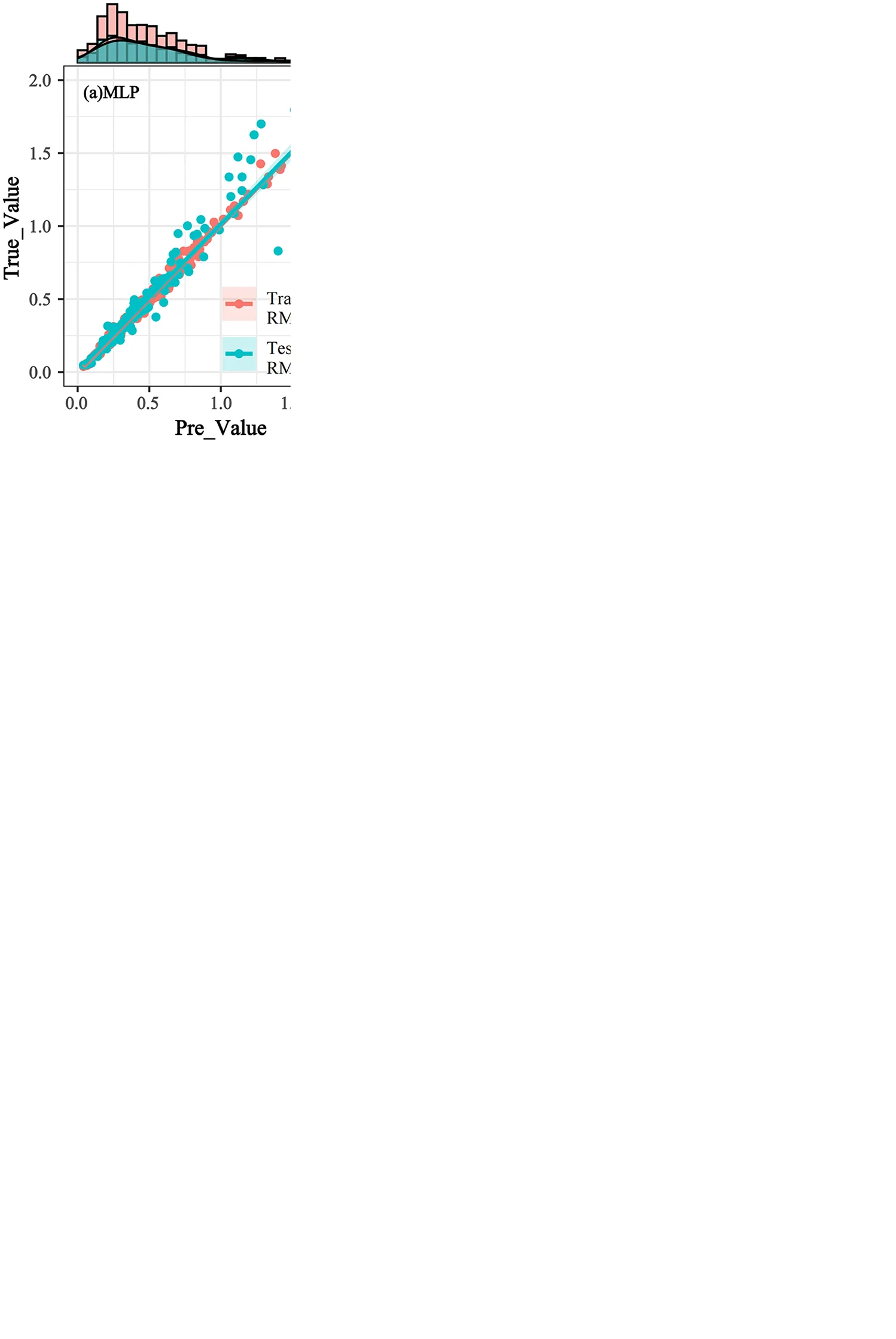
Scatter plots and marginal density distributions.

XGBoost exhibits the best overall performance, achieving the highest prediction accuracy among all models (Training set R^2^ = 0.98, RMSE = 0.06; Test set R^2^ = 0.95, RMSE = 0.08) while maintaining an extremely low level of overfitting. So XGBoost is the most reliable and stable method among the six evaluation models.

In order to demonstrate the generalization ability of the XGBoost model on out-of-sample data, the method of time division and province division was adopted for verification. In the time division, 279 samples from 2010 to 2018 were used as the training set, and 124 samples from 2019 to 2022 were used as the test set. In the province division method, a fixed province approach was used, that is, the sample data from the same province from 2010 to 2022 would not be split. At the same time, 100 times of classic Bootstrap resampling at the provincial level was used, and the out of band (OOB) was used for testing. The parameter settings of the 6 models were basically the same as those of the previous basic models. The verification results are shown in Table 5.

**Table 5:** Out-of-sample generalization performance under temporal and province-wise validation.

Type	Model	KNN	LM	MLP	RF	SVM	XGBoost
Province division	R^2^ Mean	0.398	0.740	0.647	0.610	0.746	0.701
	R^2^ std	0.413	0.220	0.277	0.122	0.221	0.085
	RMSE Mean	0.277	0.176	0.201	0.247	0.175	0.221
	RMSE std	0.077	0.050	0.052	0.109	0.053	0.108
	MAE Mean	0.172	0.132	0.157	0.173	0.132	0.147
	MAE std	0.049	0.040	0.041	0.041	0.039	0.039
Time division	R^2^	0.808	0.081	−1.818	0.531	−1.172	−0.544
	RMSE	0.067	0.146	0.257	0.105	0.225	0.190
	MAE	0.051	0.122	0.160	0.085	0.139	0.147
	MSE	0.004	0.021	0.066	0.011	0.051	0.036

In the time division method. The performance of XGboost is excellent and significantly outperforms the other models. The R^2^ value is 0.8 and the RMSE is only 0.06. In the province division verification, although the XGBoost model does not perform the best in the average R2 value, which is only 0.701, lower than LM and SVM, the variance of R^2^ is the lowest, only 0.085, indicating the stability of the XGBoost model. Moreover, the RMSE is only 0.22. The reasons for these differences are the significant differences in indicators among different provinces, especially in the eastern and western regions, which are very obvious, causing differences in the performance of the model. Overall, the time-based and province-wise validation results provide additional evidence that the XGBoost model maintains relatively strong and stable predictive performance under both temporal and geographic out-of-sample settings.

#### Stability testing and sensitivity analysis.

The stability test and sensitivity analysis were conducted using k-fold cross-validation and variable perturbation methods respectively. The test results are shown in Table 6. The stability test adopted three cross-validation methods: 3-Fold, 5-Fold, and 10-Fold, to verify that the XGBoost model is stable under different data partitioning conditions. The R^2^ values of the three cross-validation methods (3-Fold, 5-Fold, 10-Fold) were all greater than 0.96, and the mean R^2^ value of the 10-fold cross-validation was 0.98, slightly higher than that of the 3-Fold and 5-Fold. This might be because the training set samples in the 10-fold validation method were larger. The mean values of the other evaluation indicators RMSE, MSE, and MAE were relatively low, all below 0.1. The variance was also very small, below 0.035, and the maximum RMSE variance was only 0.0328. Therefore, the consistent predictive performance across the three cross-validation schemes indicates that the XGBoost model is relatively robust to different data partitioning schemes and maintains stable predictive performance across different validation subsets.

**Table 6:** Result of stability testing and sensitivity analysis.

Type	Indicator	3-Fold		5-Fold		10-Fold	
		Mean	Variance	Mean	Variance	Mean	Variance
Stability Testing	RMSE	0.0897	0.0328	0.0897	0.0328	0.0623	0.0213
	MSE	0.0119	2*e^-5^	0.0119	2*e^-5^	0.0077	5*e^-5^
	MAE	0.0481	7*e^-5^	0.0481	7*e^-5^	0.0392	0.0030
	R^2^	0.9609	0.0140	0.9609	0.0140	0.9819	0.0076
**Type**	**Indicator**	**0.05 Level**			**0.1 Level**		
		**Mean**	**Variance**	**Optimal**	**Mean**	**Variance**	**Optimal**
Sensitivity Analysis	RMSE	0.0706	0.033	0.0719	0.0823	0.0346	0.0789
	MSE	0.0067	1.3*e^-5^	0.0051	0.0078	2*e^-5^	0.0062
	MAE	0.0466	0.0115	0.0459	0.0525	0.0102	0.0419
	R^2^	0.9772	0.0142	0.9832	0.9692	0.0094	0.9782

Sensitivity analysis was conducted using variable perturbation methods at the 0.05 and 0.1 levels, respectively. And compared the mean, variance, and optimal values of four evaluation indicators. The mean values of RMSE and MAE are both less than 0.1, and the variances are both less than 0.05. The R^2^ mean is 0.97 in the 0.05 level disturbance and 0.96 in the 0.1 level disturbance, both showing excellent performance. These have verified that XGBoost has good anti-interference ability.

### Nonlinear effect between key factors and RECC

SHAP analysis is intended to interpret the predictive behavior of the XGBoost model rather than to establish causal relationships. Specifically, the SHAP value of a variable represents its model-based contribution to the predicted RECC relative to the model’s baseline prediction, conditional on the other variables included in the fitted model. Therefore, positive or negative SHAP values indicate the direction of the variable’s contribution to model predictions, rather than a causal effect on RECC. Similarly, the nonlinear patterns identified by SHAP dependence plots and the interaction patterns identified by SHAP interaction analysis should be interpreted as model-based nonlinear associations and conditional contributions.

#### Feature importance.

We used the XGBoost model combined with SHAP (Shapley Additive Interpretation) method to quantify the impact of each factor on RECC index. The global SHAP summary chart shows the average impact of each variable (Fig 5a), sorted in descending order of importance. The top eight factors are: total discharge of pollutants in wastewater(*X*_2_, SHAP = 0.156); industrial sulfur dioxide emissions (*X*_3_, SHAP = 0.104); total water consumption (*X*_1_, SHAP = 0.063); total water resources (*X*_7_, SHAP = 0.033); per capita garbage disposal capacity (*X*_14_, SHAP = 0.032); total energy production (*X*_11_, SHAP = 0.029); annual average concentration of PM2.5 (*X*_4_, SHAP = 0.022); per capita green area (*X*_9_, SHAP = 0.012). indicates that environmental indicators play a dominant role in the model.

**Fig 5:**
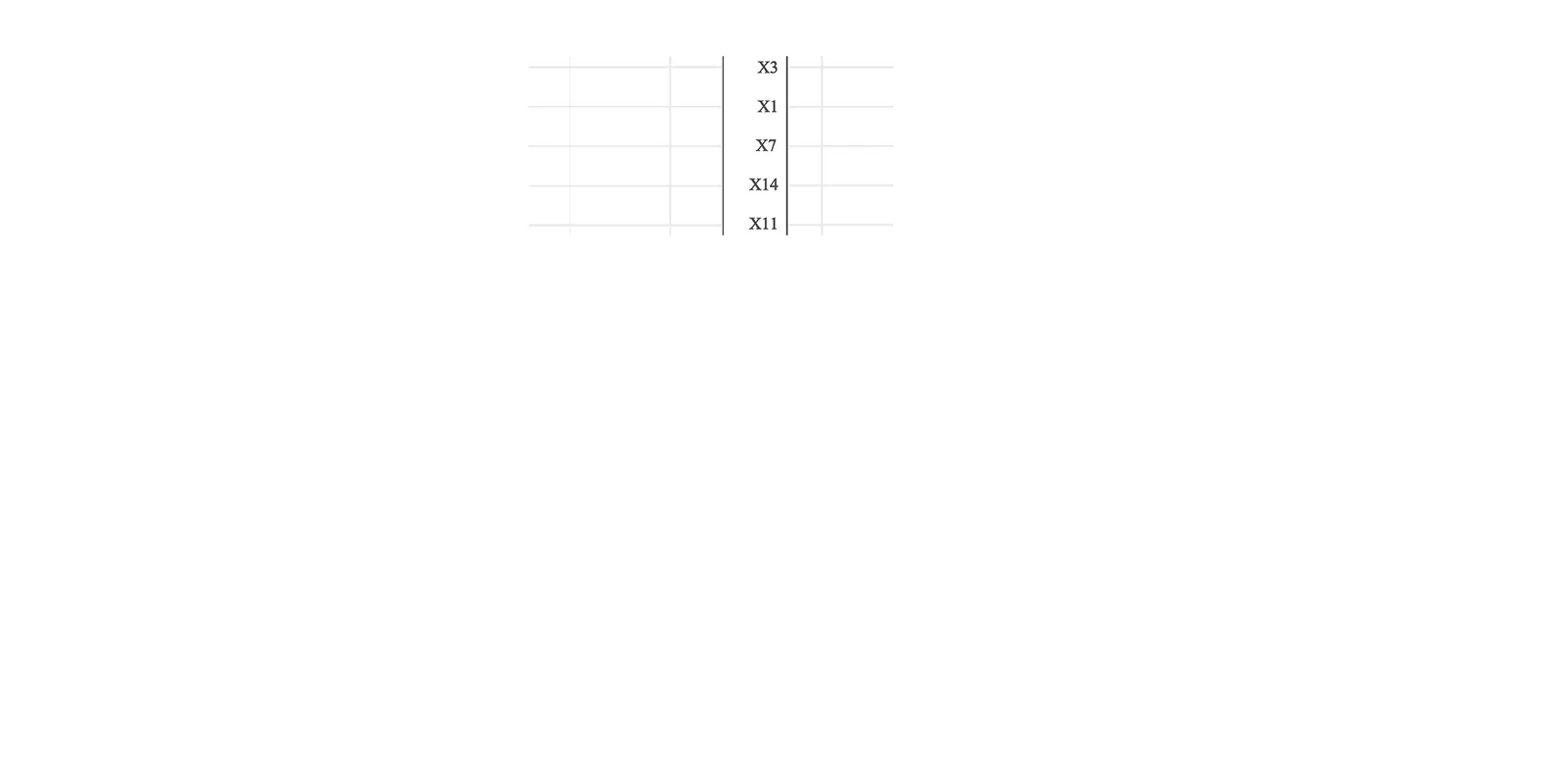
Variable SHAP value statistics chart. (a) Importance ranking, (b) SHAP summary plot.

To validate the robustness of each variable’s contribution to the model, we adopted the bootstrap resampling method (200 times) used in the XGBoost model. As shown in Table 7, the SHAP statistics of the top six key variables demonstrated overall good stability, with confidence interval widths for all not exceeding 0.05 at the 95% level. *X*_2_ had the highest average SHAP value of 0.127, 95% CI is [0.103, 0.152]) and contributed 27.78%, indicating its dominant role in model prediction. *X*_3_ had an average SHAP value of 0.100, contributing 21.88% 95% CI is [0.071, 0.126], also showing high importance. Together, *X*_2_ and *X*_3_ accounted for 49.67% of the total contribution, suggesting they are the most significant explanatory variables in the RECC prediction model. In contrast, *X*_1_ contributed 13.04%, while *X*_4_, *X*_14_, and *X*_7_ contributed 5.99%, 5.59%, and 4.43%, respectively, all with relatively low average SHAP values.

**Table 7:** Robustness test of |SHAP| and collinearity test of the top 6 variables’ |SHAP|.

Variable	Collinearity test		Robustness test of |SHAP|				
	VIF	Collinearity level	Mean	Std	95% CI Lower	95% CI Upper	Contribution(%)
X2	1.580	Low	0.127	0.013	0.103	0.152	27.785
X3	3.389	Low	0.100	0.016	0.071	0.126	21.887
X1	2.876	Low	0.060	0.007	0.047	0.069	13.043
X7	1.858	Low	0.020	0.003	0.014	0.025	4.430
X14	3.218	Low	0.026	0.009	0.014	0.043	5.597
X4	3.568	Low	0.028	0.008	0.018	0.042	5.998

Furthermore, from the perspective of different subsystems, their contributions are also very uneven. The majority of variables ranked first come from the stress subsystem, which together contribute about 80% of the influence. This indicates that at the result of model prediction, the main factor determining the carrying capacity of a region’s resources and environment is how much pressure it bears. Therefore, if we want to effectively improve the carrying capacity, the most effective way is to reduce pressure, such as strictly controlling sewage discharge and reducing the release of industrial pollutants such as sulfur dioxide.

To further analyze whether each influence factor contributes positively or negatively to the RECC index, a SHAP dependency graph of the influence factors was plotted (Fig 5b). Each point represents a sample, and the color from blue to red indicates that the value of each factor is increasing from small to large. The horizontal axis represents the SHAP value that affects the XGBoost model prediction. Using 0 as the boundary point, a SHAP value greater than 0 indicates that the factor has a positive impact on the RECC index. Conversely, the feature has a negative impact on the RECC index. The results indicate that *X*_2_, *X*_3_, and *X*_1_ have a positive contribution to the RECC index and have a wide range of effects, with SHAP values ranging from −0.15 to 0.35. *X*_7_, *X*_14_, *X*_11_ have a negative contribution to the RECC index. The impact of other variables is relatively small, and the SHAP value is about 0.

#### Nonlinear effect of single influence factor.

Firstly, to avoid issues of correlation and collinearity among the variables, the correlations of the Top 6 variables were calculated, and a collinearity test was conducted on them to prevent differences in the contribution of the variables to the model due to multiple collinearity and interdependence problems. The collinearity test in Table 7 indicates that the VIF collinearity tests of the first 6 variables are all at the Low level, and the maximum VIF value does not exceed 3.6. Fig 6 shows the Spearman and Person correlation heat map of the first 6 variables, which makes it easy to notice that the correlations among the variables are relatively weak. Only the correlation coefficient between X3 and X4 is greater than 0.5, and the correlation coefficients between the other variables are all less than 0.35. All these confirm that the 6 important variables analyzed by SHAP do not have double collinearity or interdependence among the variables.

**Fig 6:**
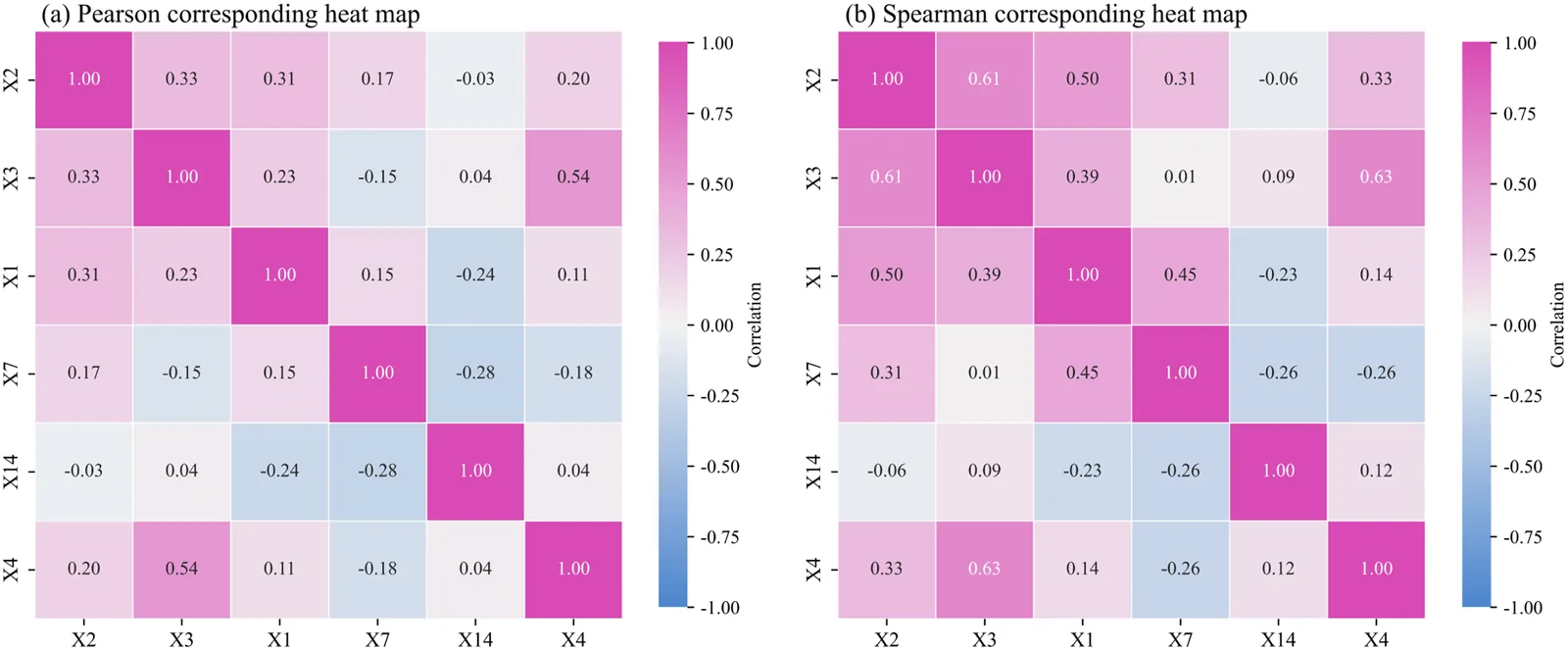
Correlation among the top 6 variables.

Then, we selected the six most important variables in the model and plotted their SHAP dependency graphs (Fig 7), to gain a clearer understanding of how key influence factors affect RECC index nonlinearly. These graphs intuitively demonstrate the marginal contribution of each variable to RECC index at different values, that is, how much impact it will have for every slight change. It was found that this effect is often not uniform, but has obvious inflection points or thresholds. After passing a certain value, the effect will suddenly become stronger, weaker, or even reverse.

**Fig 7:**
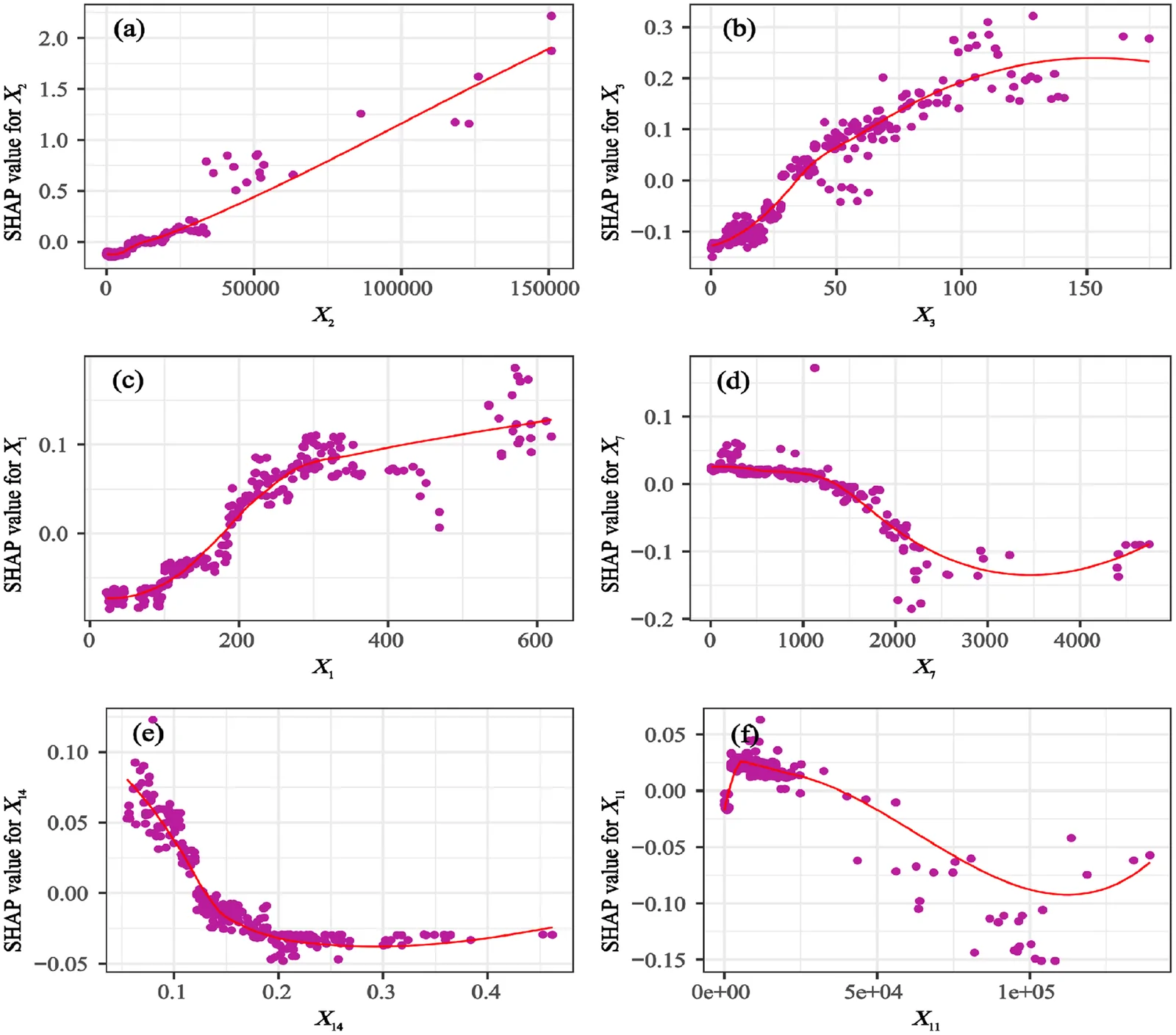
SHAP dependence plot of Single factor.

There is a turning point in the contribution of *X*_2_ (total pollutant emissions in wastewater) to RECC index (Fig 7a). When *X*_2_ is below about 50000 tons, its contribution is minimal, and the SHAP value fluctuates between 0 and 0.5; Once it exceeds 50000 tons, the SHAP value rapidly increases, and even exceeds 1.5 when it reaches around 150000 tons. This indicates that higher emissions will lead to a significant increase in RECC index of model prediction. At the same time, this threshold effect also demonstrates stability in the Bootstrap test, with a 95% confidence interval of [45308, 52557].Therefore, we must be particularly vigilant in terms of policies. When the emissions exceed 50000 tons, we must quickly strengthen sewage treatment and control new emissions, otherwise it is easy for the carrying capacity to exceed the red warning line. The impact of *X*_3_ (industrial sulfur dioxide emissions) shows a characteristic of first being fast and then slow (Fig 7b): when *X*_3_ increases from 0 to around 1 million tons, the SHAP value rapidly rises to 0.25–0.3. But after exceeding one million tons, the curve almost flattens, and no matter how many rows are added, there is no significant benefit. This indicates that there is a marginal effect on the contribution of *X*_3_ to the RECC index. After exceeding one million villages, the impact of increased emissions reaches its upper limit.This marginal effect also exhibits excellent stability. The 95% confidence interval after 200 bootstrap sampling is only [98.3, 104].

The impact of *X*_1_ (total water consumption) shows an “S” shaped trend of change (Fig 7c). When the annual water consumption of a region is less than 20 billion cubic meters, it has almost no effect on RECC index of model prediction, and the SHAP value is close to zero. This indicates that the water usage level is roughly sufficient to maintain basic production and living needs. But once the water usage enters the range of 20–40 billion cubic meters, as the water usage increases, the carrying capacity index will also rise rapidly.

*X*_7_ (total water resources) and *X*_11_ (total energy production) show negative contributions, accompanied by nonlinear characteristics (Fig 7d and f). When the total amount of water resources is below 100 billion cubic meters, the SHAP value approaches zero, but sharply drops to about −0.15 at around 200 billion cubic meters and then stabilizes. This indicates that the total amount of water resources is 100 billion cubic meters, which is the basic demand for regional resources and environment. When it is less than 200 billion cubic meters, an increase in *X*_7_ will reduce carrying capacity index of model prediction. However, after exceeding 200 billion cubic meters, the impact of an increase in *X*_7_ is limited. *X*_14_ (per capita garbage disposal capacity) shows a negative contribution, accompanied by linear characteristics (Fig 7e). When *X*_14_ is about 0.1 tons/person, the SHAP value is about 0.1. As *X*_14_ increases to 0.2 tons/person, the SHAP value steadily decreases to near zero. This indicates that the per capita garbage disposal capacity has a significant negative contribution to the RECC index of model prediction, and increasing it can greatly enhance the carrying capacity. However, after exceeding 0.2 tons/person, this impact tends to stabilize.

Overall, factors that affect the carrying capacity of resources and environment are rarely linear or singular. They often have the strongest effect within a specific numerical range, and after passing the threshold, they saturate, invert, or even deteriorate. Therefore, the key to precise governance is not simply to reduce pollution or increase resources, but to identify the sensitive range of each variable.

#### Interaction influence among key influence factors.

To further investigate the mechanisms underlying the nonlinear relationships identified by the XGBoost-SHAP model, we examined the pairwise interactions among six key influence factors (Fig 8). Rather than interpreting the SHAP interaction patterns as causal effects, these results describe how the contribution of one factor to the model-predicted RECC varies conditional on the level of another factor. The interaction patterns nevertheless provide useful evidence for identifying potential synergistic and antagonistic relationships that are consistent with the pressure–state–response structure of resource and environmental carrying capacity.

**Fig 8:**
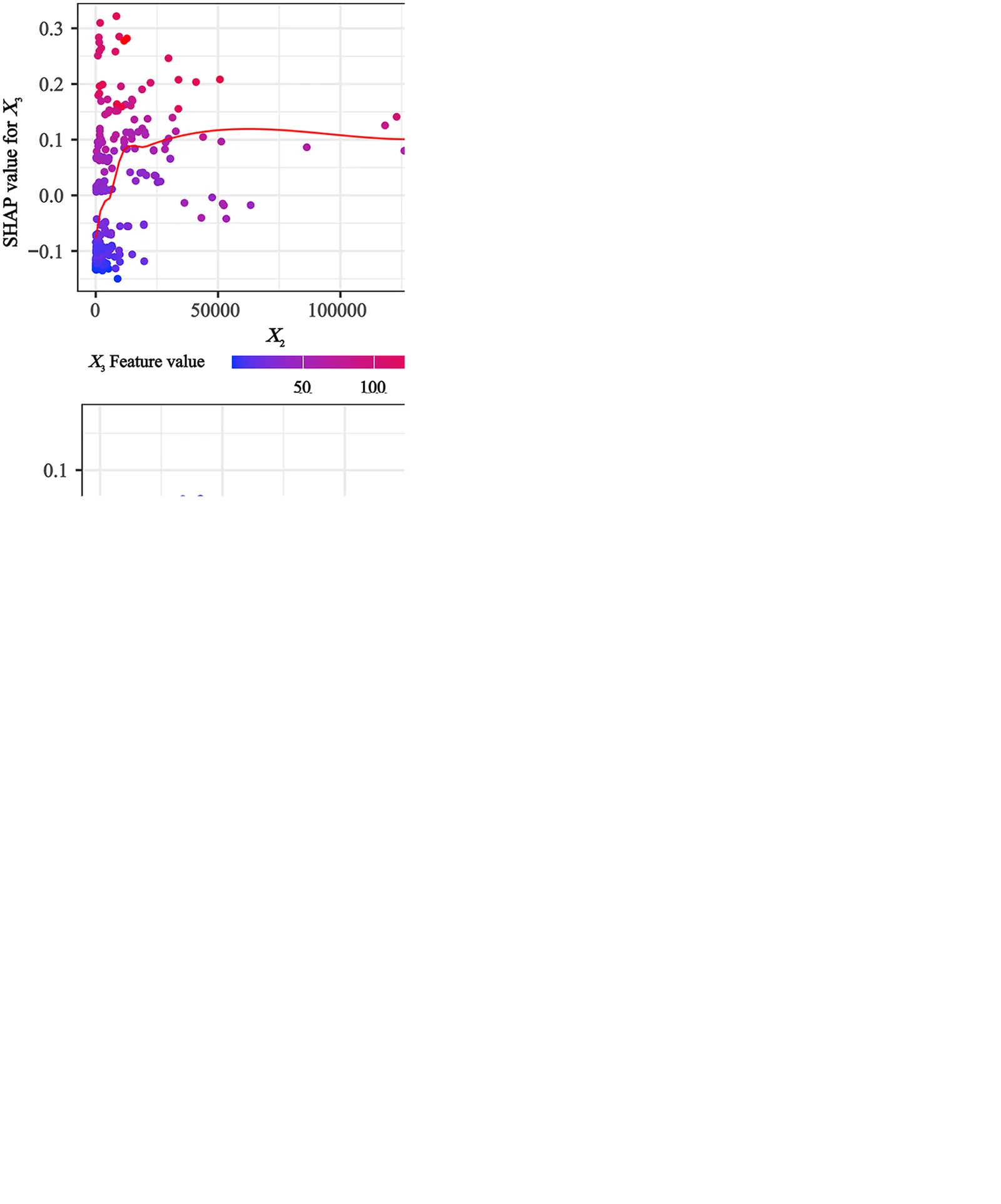
SHAP interaction dependency plots.

A clear positive interaction is observed between *X*_2_ (total pollutant discharge in wastewater) and *X*_3_ (industrial sulfur dioxide emissions) (Fig 8a). As *X*_2_ increases, the positive contribution associated with *X*_3_ becomes stronger, particularly at relatively high levels of *X*_2_. This pattern can be understood from the common pollution-pressure mechanism underlying the two indicators. Wastewater pollutant discharge and industrial sulfur dioxide emissions represent different forms of environmental pressure generated by production and consumption activities. Their simultaneous variation may therefore reflect the intensity and concentration of regional industrial activities and associated environmental pressures. When multiple pollutant pressures increase concurrently, the capacity of environmental systems and pollution-control infrastructure to absorb and process these pressures can become increasingly constrained.

A similar positive interaction is observed between *X*_2_ and *X*_1_ (Fig 8b). The contribution associated with *X*_1_ becomes increasingly positive when *X*_2_ reaches a higher level. *X*_1_ represents total water consumption, and its interaction with wastewater pollutant discharge can be interpreted through the coupled water–environment system. Water consumption is not only a resource-use variable but is also closely connected with wastewater generation, treatment demand, and the pressure placed on regional water-environment systems. Previous analysis in this study shows that *X*_1_ has an S-shaped relationship with the RECC index, with its contribution increasing rapidly within an intermediate range of water use. Therefore, the positive *X*_1_–*X*_2_ interaction may reflect the fact that the environmental implications of water use depend on the simultaneous level of pollutant pressure. Under relatively high wastewater pollutant discharge, improvements in water-use efficiency, wastewater treatment, and water-resource management can generate larger improvements in the modeled carrying-capacity state.

In contrast, *X*_7_ (total water resources) exhibits an antagonistic interaction with *X*_2_ (Fig 8c). The negative interaction does not necessarily imply that greater water availability directly improves the environmental consequences of pollution. Instead, it indicates that the marginal contribution of *X*_2_ to the model-predicted RECC becomes weaker when regional water-resource endowment is high. This pattern is consistent with the buffering role of natural resource endowment in regional resource–environment systems. Regions with relatively abundant water resources may possess greater capacity to absorb short-term resource pressures and maintain production and ecological functions. However, such buffering capacity should not be interpreted as an unlimited ability to offset pollution. Indeed, the univariate SHAP analysis shows that *X*_7_ itself has a nonlinear negative contribution, with its effect becoming relatively stable after a high-resource threshold is reached. This suggests that water-resource abundance may modify the sensitivity of RECC to pollution pressure, but cannot substitute for direct pollution reduction or environmental management.

The interaction involving *X*_14_ (per-capita waste-disposal capacity) provides another example of a response-capacity mechanism. *X*_14_ has a relatively weak overall contribution to the prediction, but its interaction with *X*_2_ and *X*_7_ indicates that waste-management capacity can modify the effects of environmental pressure and resource conditions (Fig 8d–e). This result is consistent with the PSR framework adopted in this study, in which response capacity represents the ability of regional systems to alleviate or manage environmental pressures. The nonlinear pattern observed in the univariate SHAP analysis further indicates that the contribution of *X*_14_ becomes relatively stable after approximately 0.2 tons/person. Thus, improvements in waste-treatment capacity may have stronger effects when the existing response capacity is relatively limited, whereas additional capacity beyond a certain level may generate diminishing returns. The interaction results imply that the effectiveness of response capacity depends partly on the prevailing resource and environmental pressures rather than being independent of them.

The *X*_14_–*X*_11_ interaction presents a different pattern (Fig 8f). *X*_1_ represents total energy production and has a predominantly negative contribution to the RECC index in the univariate analysis. When *X*_14_ reaches a relatively high level, the negative contribution associated with *X*_1_ becomes more pronounced. This pattern may reflect the structural characteristics of energy-intensive regional production. Higher waste-disposal capacity does not necessarily offset the environmental pressures associated with energy production. Instead, when energy production is accompanied by intensive industrial activity, improvements in end-of-pipe waste-management capacity may be insufficient to compensate for the broader resource consumption and environmental pressures generated by energy production. In this sense, the *X*_14_–*X*_11_ interaction suggests a distinction between pollution-response capacity and the underlying production structure: improving treatment capacity can reduce specific environmental pressures, but it does not necessarily eliminate the environmental burden associated with resource- and energy-intensive development.

Overall, the interaction analysis reveals that the RECC system is characterized by both synergistic and antagonistic relationships. *X*_3_ and *X*_1_ amplify the contribution of *X*_2_ under relatively high pollution-pressure conditions, whereas *X*_7_ modifies and weakens the marginal contribution associated with *X*_2_. *X*_14_ further demonstrates that response capacity interacts with both resource availability and energy-production pressure rather than exerting an independent effect. These findings are broadly consistent with the integrated nature of the PSR framework, in which environmental pressure, resource conditions, and societal response jointly determine regional carrying capacity. Previous studies have emphasized that RECC is a coupled resource–environment system in which water resources, pollution pressure, and management capacity interact rather than operate independently. Therefore, the SHAP interaction results are regarded as evidence of the nonlinearity of the model. These conclusions are in line with the known environmental processes rather than direct causal effects. This is because SHAP can explain the decision-making structure of the predictive model, but it cannot establish causal relationships on its own. This is also a limitation of this study.

## Discussion

This study innovatively proposes an interpretable machine learning method, which combines XGBoost and SHAP to analyze how the RECC of various provinces in China has changed from 2010 to 2022, and quantify its driving effects. This framework can not only handle the complex nonlinear relationships in the Pressure Support Regulation (PSR) system, but also quantify the impact of individual influence factors and how they complement or cancel each other out. In this way, the driving mechanism of RECC is no longer vague, but becomes clear and measurable, pushing forward research in this field.

Firstly, research has found that the overall resource and environmental carrying capacity of the country has significantly increased in the past decade, which is consistent with the country’s overall direction of promoting green development and building ecological civilization – especially since the release of the “Opinions of the State Council on Accelerating the Construction of Ecological Civilization” in 2015, the effect has gradually become apparent. Previous studies static indicators are mostly used to compare the carrying capacity of different regions [30,31]. However, through a dynamic perspective of time and space, we not only see the overall trend, but also understand which path each region is taking. For example, the eastern coastal provinces have recovered particularly quickly, precisely because of strict environmental regulations and advanced economic structures, which have formed a positive cooperation between the two. This also indicates that it is not enough to simply study who is higher and who is lower at a certain point in time. It is necessary to extend the timeline in order to truly understand how sustainable development is implemented in different places.

In terms of methodology, The objective of the XGBoost-SHAP framework is not to validate the entropy-TOPSIS evaluation results. Instead, it aims to uncover nonlinear relationships, marginal effects, and interaction patterns among the drivers associated with the RECC index. Meanwhile, the performance of XGBoost once again proves that decision tree based models are indeed adept at handling the complexity of environmental systems [32]. Although this type of machine learning model predicts very accurately and fits the RECC index well, it has always been criticized for not being able to explain the reasoning clearly and only knowing the results without knowing why. To address this issue, we incorporated the SHAP method into the model, opening a window into the black box of XGBoost and clearly demonstrating how each variable affects the final result. This responds to the concerns of many scholars that although machine learning can judge good from bad, if it cannot explain the reasons, it will be difficult for policy makers to act based on it [33,34]. Our framework precisely fills this gap, retaining the high precision of machine learning while bringing interpretability that is only available with traditional statistical methods. For example, analysis has found that industrial pollutant emissions (*X*_2_, *X*_3_) are key factors affecting RECC, which is consistent with previous research that emphasized the importance of the pressure subsystem [35,36]. But the difference is that traditional methods can only generalize their importance, while our model can not only draw their nonlinear impact curves on bearing capacity, but also identify specific critical values, such as when *X*_2_ exceeds 50000 tons and *X*_3_ exceeds 1 million tons, the bearing capacity will significantly decrease. This precise quantitative data is much more practical than traditional qualitative sorting can directly guide policy-making [37,38]. Furthermore, we also discovered the interactive relationship between influence factors. Although frameworks like DPSIR theoretically acknowledge that subsystems can interact with each other, few studies have truly quantified this interaction. For example, there is a significant synergistic effect between *X*_2_ and *X*_3_, and controlling both pollutants together yields better results. There is an antagonistic effect between the total amount of water resources (*X*_7_) and pollution (*X*_2_), where the more water there is, the less damage caused by pollution. These hidden interaction patterns, which have been almost unexplored in previous research, are particularly valuable for scientifically formulating environmental protection strategies.

Of course, this study also has its limitations. Firstly, we are using provincial data, which may overlook the differences between different cities or counties within the province. If we can use city, county, or even finer grained data in the future, the conclusions will be more accurate. Secondly, although the PSR framework is already quite comprehensive, it may miss some important factors such as technological innovation capability, government governance level, and public environmental awareness. In the future, we can try to build a more complete indicator system. Thirdly, the causal interpretation of the machine-learning results, although XGBoost combined with SHAP can effectively characterize nonlinear predictive associations, variable contributions, and conditional interactions, these results do not by themselves establish causal relationships. The observed SHAP patterns may reflect correlations arising from omitted variables, reverse relationships, common trends, or other forms of confounding. Therefore, the identified nonlinear and interaction patterns should be regarded as model contribution, rather than definitive causal effects. Future research could combine the explainable machine learning framework with causal inference approaches, such as causal forests, double machine learning, or other identification strategies, to further investigate the causal effects of key factors on RECC.

## Conclusion

This paper analyzes the evolution of resource and environmental carrying capacity trends from 2010 to 2022 from a provincial perspective in China. An explainable machine learning model based on XGBoost and SHAP was employed to examine the driving mechanisms. Several significant conclusions were drawn.

(1) Resource and environment carrying capacity levels across all Chinese provinces had returned to safe and sustainable levels by 2022. The average carrying capacity index decreased from 1.18 in 2010 to 0.39 in 2022. The Southwest and Northwest regions exhibited higher carrying capacity levels than other areas, while the Eastern and Central regions initially showed weaker capacity but subsequently strengthened rapidly.
(2) The XGBoost model outperformed other machine learning models. On the training and testing datasets, R^2^ values reached 0.98 and 0.95 respectively, with RMSE values of 0.06 and 0.08. Additionally, the XGBoost model demonstrated excellent stability. Using k-fold cross-validation and variable perturbation, the model maintained R^2^ values above 0.95 and RMSE below 0.1.
(3) The XGBoost-SHAP model indicates that *X*_2_ (total pollutant discharge in wastewater) and *X*_3_ (industrial sulfur dioxide emissions) are the most critical drivers for model contribution, with SHAP values of 0.156 and 0.104, respectively. *X*_2_ exhibits a significant positive correlation with the RECC index predicted by the model, and this influence significantly intensifies when *X*_2_ exceeds 50,000, indicating a clear threshold effect. *X*_3_ exhibits a nonlinear positive correlation with the RECC index, featuring a marginal effect. A value of 100 for *X*_3_ serves as the marginal threshold, beyond this level, the impact ceases to increase.
(4) The interaction effects among driver factors exert a strong nonlinear influence on the RECC index. *X*_3_ and *X*_1_ promote the positive effect of *X*_2_ and amplify its marginal effect, with SHAP values increasing by up to 0.1 at high interaction levels. Conversely, *X*_7_ and *X*_14_ significantly inhibit the positive effect of *X*_2_, reducing SHAP values by 0.05 at maximum.

## Supporting information

S1 Dateset Original data. (XLSX)
